# Nuclear Import of Yeast Proteasomes

**DOI:** 10.3390/cells4030387

**Published:** 2015-08-07

**Authors:** Julianne Burcoglu, Liang Zhao, Cordula Enenkel

**Affiliations:** Biochemistry Department, University of Toronto, Toronto, ON M5S 1A8, Canada; E-Mails: j.burcoglu@mail.utoronto.ca (J.B.); liangfrank.zhao@utoronto.ca (L.Z.)

**Keywords:** proteasome, nuclear transport, importin, karyopherin, Blm10, proteasome granules

## Abstract

Proteasomes are highly conserved protease complexes responsible for the degradation of aberrant and short-lived proteins. In highly proliferating yeast and mammalian cells, proteasomes are predominantly nuclear. During quiescence and cell cycle arrest, proteasomes accumulate in granules in close proximity to the nuclear envelope/ER. With prolonged quiescence in yeast, these proteasome granules pinch off as membraneless organelles, and migrate as stable entities through the cytoplasm. Upon exit from quiescence, the proteasome granules clear and the proteasomes are rapidly transported into the nucleus, a process reflecting the dynamic nature of these multisubunit complexes. Due to the scarcity of studies on the nuclear transport of mammalian proteasomes, we summarised the current knowledge on the nuclear import of yeast proteasomes. This pathway uses canonical nuclear localisation signals within proteasomal subunits and Srp1/Kap95, and the canonical import receptor, named importin/karyopherin αβ. Blm10, a conserved 240 kDa protein, which is structurally related to Kap95, provides an alternative import pathway. Two models exist upon which either inactive precursor complexes or active holo-enzymes serve as the import cargo. Here, we reconcile both models and suggest that the import of inactive precursor complexes predominates in dividing cells, while the import of mature enzymes mainly occurs upon exit from quiescence.

## 1. Introduction

Proteostasis is defined as a series of biological processes that work in concert to create and maintain a functional proteome in a living cell. These processes involve protein synthesis, folding, trafficking, disaggregation, and degradation in response to intracellular and extracellular demands. For a long time the need for intracellular protein degradation was not realised because it was deemed counterintuitive that proteins, which are synthesised at the expense of energy, would be targeted for destruction. This paradigm was shifted upon the discovery of ATP-dependent proteolysis, a process that removes proteins which regulate a wide variety of fundamental cellular processes and need to be eliminated in time [1].

In the 1980s, Hershko and Ciechanover discovered polyubiquitin, a chain of highly conserved 76-amino acid polypeptides which, when covalently linked to proteins, marks them for degradation by ATP-dependent proteolysis [2]. At the same time, Wilk and Orlowski isolated a 700 kDa multisubunit protease complex, later named the proteolytic core (CP) of the proteasome, as the key enzyme of the ATP- and ubiquitin-dependent proteolytic system (UPS) [3].

The UPS achieves ~90% of protein breakdown in growing yeast and cultured mammalian cells. Prominent examples of UPS substrates are cell cycle regulating proteins such as cyclins, cyclin-dependent kinases and their inhibitors, tumour suppressor protein p53, IκBα, transcriptional factors, and misfolded and malfunctioning proteins [4,5].

As the proteasome is the key protease of the UPS, it is noteworthy to shed light on the cellular compartments where proteasomal proteolysis is mainly required and to analyse intracellular dynamics of the proteasome. Almost all literature describes proteasome dynamics in dividing cells, though they comprise only a small fraction of our body’s cells.

In the 1980s, Franke and colleagues, and later Mikecz and colleagues, localized proteasomes mainly to the nucleus of dividing vertebrate cells, as shown by indirect immunofluorescence and immunogold electron microscopy [6,7,8,9]. Mikecz’s contribution was that proteasomal protein degradation is an intrinsic function of the nucleus. Nuclear proteasomes were found to be proteolytically active by degrading proteins in focal subdomains in the nucleus of human cells [10]. With the discovery of the green fluorescent protein (GFP) in 1995, direct fluorescence microscopy of living cells expressing GFP-labelled reporter proteins became the state of the art for localisation studies [11]. Especially yeast, a prime model organism of eukaryotic cells, proved to be suited for GFP-labelling techniques, since endogenous proteins can be functionally replaced by their GFP-tagged versions using homologous recombination into the chromosomal locus. By this manner, GFP-tagged proteasomal reporter proteins are expressed behind the endogenous reporter and fully incorporated into the proteasome complex. Almost each subunit of the yeast proteasome can be functionally tagged with GFP. Without previous limitations given by ambiguous antibodies and fixation conditions used for indirect detection methods, live cell imaging revealed a substantial localisation of GFP-tagged proteasomes within the nucleus throughout the cell cycle in yeast [12,13,14,15]. When mammalian subunits of the proteasome are tagged with GFP, complete incorporation of the fusion protein into the proteasome complex is partly achieved due to the presence of endogenous subunits. Nevertheless, thorough localisation studies using GFP-technologies in proliferating human melanoma cells (Mel JuSo) confirmed the predominant nuclear localisation of proteasomes labelled with GFP-tagged CP subunit α4 [16]. Though in human fibrosarcoma cells (HT1080), immune-specific isoforms of the CP labelled by GFP-tagged β1 seemed to be equally distributed between the nucleoplasm and cytoplasm [17].

In contrast to these studies, CP subunit β7 labelled with GFP exclusively localised to the cytoplasm of HeLa cells. Upon DNA damage, the GFP-tagged proteasomes were reported to be nuclear [18].

The question arose as to whether the predominant nuclear localisation of proteasomes pointed to a major site of proteolysis in the nucleus. Does the nuclear localisation of proteasomes imply that nascent misfolded proteins are imported into the nucleus for proteasomal degradation? There is evidence in the literature accounting for this possibility, at least that the degradation of cytosolic proteins requires their nuclear import [19,20,21,22]. However, there is also compelling evidence that the degradation of short-lived nuclear proteins depends on their nuclear export into the cytoplasm, suggesting a high traffic volume of protein substrates between the nucleoplasm and cytoplasm [23]. With a doubling time of two hours in yeast, protein turnover might also be influenced by cell cycle specific effects, whereas protein stability is less affected in cultured mammalian cells with a doubling time longer than 12 hours.

In compliance with the demand for protein degradation, proteasomes seem to change their subcellular localisation between the nucleoplasm and cytoplasm. With the transition from cell proliferation to quiescence, the ATP level declines due to the shortage of nutrients [24]. In yeast, quiescence is reached when cells are grown to stationary phase. Under these conditions proteasomes accumulate at the nuclear envelope (NE)/ER membrane. However, due to the small size of the yeast nucleus, it is difficult to distinguish by fluorescence microscopy whether proteasomes accumulate at the cytoplasmic or nucleoplasmic side of the NE/ER membrane [13,15]. Electron microscopy of immunogold-labelled proteasomes in fission yeast revealed proteasome accumulation at the nucleoplasmic side of the NE [25]. Likewise, the cell density of mammalian cell lines, the cell type, and the cell cycle stage have impacts on the localisation of proteasomes when they appear to be attached to NE/ER-resident structures [26,27,28]. Thus, cultured mammalian cells also sense the shortage of nutrients, which influences proteasome localisation.

**Figure 1:**
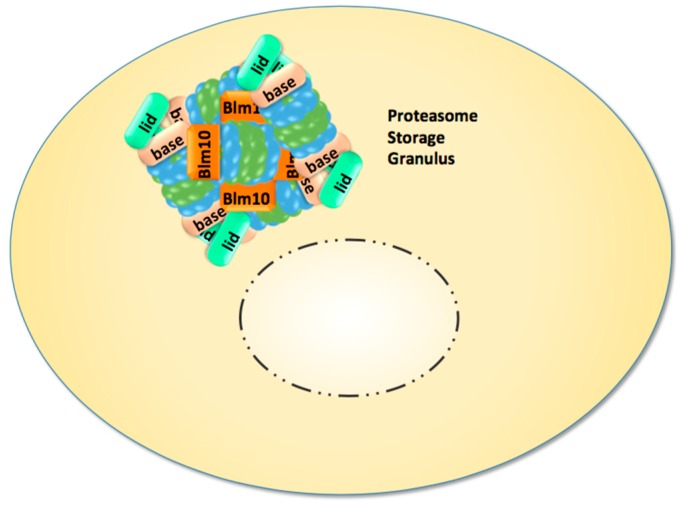
In quiescent yeast cells nuclear proteasomes are depleted and CP, RP, and Blm10-associated CP are sequestered into reversible and motile granules, which accumulate in the nuclear periphery and migrate through the cytoplasm.

Recent studies directed our attention to the enigmatic proteasome granules in the nuclear periphery of quiescent yeast and mammalian cells [29]. As mentioned above, quiescence in yeast can be induced by growth to stationary phase but also by cell cycle arrest through proteasome mutations and inhibition. Subsequently, proteasome granules arise as membraneless organelles close to the NE/ER, and seem to pinch off and migrate as stable entities through the cytoplasm (Figure 1). Cultured mammalian cells arrested in cell cycle progression by proteasome inhibition show proteasome granules as well, suggesting a conserved mechanism underlying their organisation. With the withdrawal of proteasome inhibition and the resumption of cell growth, proteasome granules rapidly clear and proteasomes are imported into the nucleus to promote cell cycle progression [29].

Proteasome granules were first described by Sagot’s laboratory to serve as storage compartments, and thus initially named proteasome storage granules (PSG) [15]. Their presence is reported to protect cells against proteo- and genotoxic stress and confers fitness during aging [30,31,32]. The PSG shares similar features with the juxta-nuclear quality compartment (JUNQ) with regards to its content of proteasomes, its reversibility, and its motility. Thus, we assume that PSG and JUNQ describe the same structure of proteasome agglomerations [31]. Apart from storage functions, the PSG/JUNQ could also point to a major site of proteasomal proteolysis in quiescence [33]. Few studies exist in primary cell lines of neurons expressing GFP-labelled proteasomes. Their dendrites harbour multiple motile proteasome granules, suggesting that proteasome granules exist in non-dividing mammalian cells, which essentially comprise the minority of our body’s cells [34].

In quiescent yeast, nuclear proteasomes are depleted with the appearance of proteasome granules at the nuclear side of the nuclear envelope [15]. How proteasome granules or single proteasomes exit the nucleus is unknown. The nuclear import of proteasomes is much better understood and we will focus on the work in yeast because nuclear transport in yeast occurs through nuclear pores due to the closed mitosis, in which the NE and the NPC remain intact. In somatic cells of higher eukaryotes, the NE completely disassembles and reassembles during open mitosis, allowing alternative nuclear uptake mechanisms for proteasomes.

## 2. Nuclear Transport of Proteasomes

### 2.1. Structure of the Proteasome

Here, we shortly summarise the structural aspects of proteasomes, which are important to understanding how proteasomes are transported through nuclear pores. For convenience, we refer to reviews for more detailed information on proteasome structure.

The proteasome is a highly conserved, ~40 subunit holo-enzyme of about 2.5 MDa and is assembled from two complexes: the core particle (CP, 20S) and the regulatory particle (RP, 19S) with molecular masses of ~700 kDa and ~950 kDa, respectively [35,36]. Proteasome holo-enzymes exist in two distinct configurations, either as single capped RP-CP or as double-capped RP-CP-RP, also called the 26S or 30S proteasomes, respectively [37]. If cells express GFP-labelled proteasomal subunits, distinct bands for GFP-labelled RP-CP and RP-CP-RP can be resolved by native polyacrylamide gel electrophoresis (PAGE) and green fluorescent protein (GFP) imaging analysis [38]. In dividing cells, RP-CP and RP-CP-RP are the predominant proteasome configurations and comprise up to 0.5% of the intracellular protein. As such, at 1–2 × 10^4^ molecules per cell, proteasomes represent the second most abundant protein complexes in yeast [39].

The CP harbours the catalytic chamber of the proteasome and resembles the barrel-shaped ancestor of prokaryotic cells, which is built by axial stacking of two outer heptameric α-rings and two inner heptameric β-rings with identical α- and β-subunits [40]. In eukaryotic cells, each α- and β-subunit is different, resulting in CP with an α_1-7_β_1-7_β_1-7_α_1-7_ configuration [41]. In principle, the CP is assembled from two CP precursor complexes, referred to as half-CP, which is composed of one α- and one β-ring, where certain β-subunits have β-pro-peptides. Five CP-dedicated chaperones, named Pac/Pba/Poc 1–4 and particularly Ump1, control the incorporation of CP subunits into the precursor complex and guide the dimerisation of the half-CP into the pre-holo-CP, an unstable intermediate of CP maturation [42,43]. Autocatalytic cleavage of the β-pro-peptides within the pre-holo-CP triggers the excavation of the central catalytic chamber and the degradation of Ump1, the first substrate of the nascent CP [44,45].

CP maturation involves major conformational changes [46]. Within CP precursor complexes, the α- and β-subunits form loosely packed rings with wider α-ring openings than in the mature CP. With the maturation of the catalytic chamber, the openings of both α-rings are tightened, resulting in mature CP with latent enzyme activity. Access through the α-ring gates is achieved by the interaction of the CP with the RP [47].

In addition to α-ring gating, the RP is responsible for delivering protein substrates into the CP [48]. The RP consists of a base and lid complex. Together they contain ~20 different subunits that are divided into two subgroups, the Regulatory particle of triple-ATPase (Rpt) subunits and the Regulatory particle of non-ATPase (Rpn) subunits. The RP lid contains at least nine Rpns and primarily functions to deubiquitylate the substrates [49,50]. The RP base contains Rpn1, Rpn2, Rpn10, and Rpn13 and the hexameric ring of Rpt1-6, which binds adjacent to the CP. Collectively, the RP base receives receptors for polyubiquitin chains, unfolds the substrate upon deubiquitylation, and translocates the unfolded polypeptide into the CP in an ATP-dependent manner [51]. To date, two different models for RP assembly have been described [52]. The first model proposes that RP assembly is independent of the CP. Accordingly, the assembly of the RP base subunits is assisted by four RP-dedicated chaperones, named Hsm3, Nas2, Nas6, and Rpn14, which are released upon the association with the RP lid. The second model favours a pathway where, in the presence of RP-dedicated chaperones, the CP serves as a template for the incorporation of RP base subunits. However, the CP template-driven model is challenged by X-ray structure analysis showing that the RP-dedicated chaperones obstruct the interface between the RP base and the CP α-ring [53]. Also, the finding that RP base and lid can be assembled from recombinant proteins lends more weight to CP-independent assembly [54].

### 2.2. The Concept of Nucleocytoplasmic Transport through the Nuclear Pore

The exchange of macromolecules between the nucleus and the cytoplasm occurs through NPCs studded throughout the NE [55,56]. The NPC can be described as a highly selective molecular sieve that is partially sealed by intrinsically disordered phenylalanine-glycine (FG)-rich nucleoporins (Nup) which prevent random diffusion of proteins larger than ~40 kDa or that have a diameter of 5 nm [57]. The translocation of larger proteins and protein complexes requires an active transport system that can overcome the diffusion barrier built by the NPC [58]. The key components of the active transport system are (i) transport signals on the cargo protein indicating the final destination—the NLS (nuclear localisation sequence) for nuclear import and the NES (nuclear export signal) for nuclear export; (ii) NLS- and NES-specific transport receptors; and (iii) the small GTPase Ran, named Gsp1 in yeast. NLS- and NES-specific transport receptors shuttle the cargo protein through the NPC. Commonly, transport receptors belong to the family of β-karyopherins, alternatively named β-importins/exportins, most of which are members of the HEAT-repeat family with an α-solenoid fold [59,60]. The directionality of nuclear transport is determined by the RanGTP/GDP gradient across the NE. Ran, named Gsp1 in yeast, exists in its GTP- and GDP-bound state in the nucleoplasm and cytoplasm, respectively, as a result of the activities of the Ran guanine nucleotide exchange factor (RanGEF) in the nucleoplasm and the RanGTPase activating protein (RanGAP) in the cytoplasm [61]. According to the well-established nuclear transport model, during import, the cargo-importin complex formed in the cytoplasm passes through the NPC into the nucleus where it encounters RanGTP. Direct binding of RanGTP to the importin triggers the release of cargo into the nucleus. Importin-RanGTP returns to the cytoplasm where hydrolysis of GTP converts RanGTP into RanGDP, which then frees the importin for the next cycle of nuclear import. During export, the cargo-exportin-RanGTP complex formed in the nucleoplasm passes through the NPC into the cytoplasm. GTP hydrolysis causes the dissociation of the export complex and thereby the release of the cargo and exportin [61].

### 2.3. The Canonical Nuclear Import Pathway

Thirteen β-karyopherins, which achieve the bulk transport of proteins through the NPC in yeast, exist [59]. The first discovered import pathway was the canonical pathway using the importin/karyopherin αβ heterodimer, named Srp1/Kap95 in yeast [62], which recognises the canonical/classical NLS (cNLS). Two types of cNLS are known: the monopartite cNLS, a cluster of basic amino acids, and the bipartite cNLS, in which two clusters of basic amino acids are spaced by about 10 amino acids. Prototypes of the monopartite and bipartite cNLS are found in the SV40 large T-antigen (PKKKRKV) and in nucleoplasmin (KR[PAATKKAGQA]KKKK), respectively [63].

Since proteasomes are imported by the canonical import pathway in yeast, we will not address the non-canonical pathways.

### 2.4. Nuclear Import of Proteasomes during Cell Division

#### 2.4.1. Nuclear Import of the CP

The ability to study the nucleocytoplasmic transport of the proteasome has been greatly enhanced by the development of live cell imaging using GFP reporter proteins and direct fluorescence spectroscopy. In yeast, GFP-labelled versions of different proteasomal subunits expressed are fully incorporated into proteasomes, and thus represent reliable reporters of the CP, RP base, and lid. Advanced fluorescence correlation spectroscopy (FCS) recently allowed the thorough examination of real-time spatio-temporal dynamics of proteasome holo-enzymes with an RP-CP-RP configuration [64].

In the past, several attempts were made to study the nuclear import of the CP, where the CP was labelled *in vitro* with fluorescein dyes and added to reconstitution systems of nuclear import in digitonin-permeabilised mammalian cells. The source of the CP was often an akaryotic cell such as *Thermoplasma acidophilum* [65]. Findings from electron microscopy studies with cNLS-coated gold particles, show that the NPC can expand to accommodate cargoes with a diameter of up to 39 nm [66]. This opening of the NPC could theoretically allow the longitudinal passage of proteasome holo-enzymes with an RP-CP-RP configuration, assuming a cylindrical shape with 20 nm diameter × 45 nm length. However, due to the permeability barrier of the NPC, the nuclear import of macromolecules such as RP-CP-RP requires an active transport system and depends on specific NLS, which are recognised by cognate transport receptors.

Putative cNLS resembling the monopartite prototype of the SV40 large T-antigen were found in distinct α-subunits of yeast and human CP. Fused to non-nuclear proteins such as fluorescein-labelled albumin, these proteasomal cNLS promoted nuclear import into digitonin-permeabilised mammalian cells, suggesting that the CP is imported by the canonical pathway [67,68]. Further *in vitro* studies revealed that cNLS mutant CP from *Thermoplasma acidophilum*, in which the cNLS of each α-subunit was genetically truncated, failed to be imported into the nucleus, suggesting that proteasomal cNLS plays a role in the nuclear import of proteasomes [69]. However, it was also observed that the nuclear import of the human CP was not stimulated by the addition of the cNLS receptor importin αβ, raising the possibility that the nuclear import of proteasomes differs from cNLS cargoes [70].

Meanwhile, it is widely accepted that the reconstitution system using digitonin-permeabilised mammalian cells has limitations. Factors such as endogenous importin αβ that promote the nuclear import of cNLS cargoes might not have been completely depleted and subsequently interfere with exogenous importin αβ. The accessibility of proteasomal cNLS within the CP also appeared to vary as suggested by Tanaka in 1990. Two conformations of the CP were proposed to exist, one with accessible cNLS and another with masked cNLS. The accessibility of the cNLS was proposed to be regulated by tyrosine phosphorylation [71]. Accordingly, purified CP may happen to be either in an import-competent state or an import-incompetent state.

**Figure 2:**
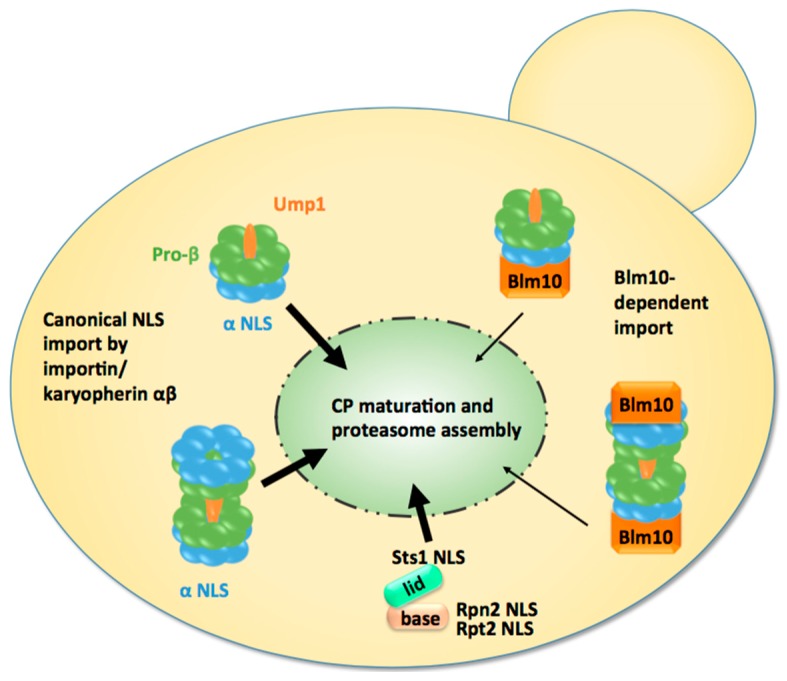
Model of nuclear import of CP precursor complexes and RP modules in proliferating yeast cells. Either the half-CP precursor or the pre-holo-CP, as indicated by the presence of immature pro-β subunits and the maturation factor Ump1, serve as the import cargo. They are mainly transported by the canonical pathway using classical nuclear localisation sequences (NLS) within α subunits and the receptor importin/karyopherin αβ. Blm10 provides an alternative import pathway. Modules of RP base and lid are imported by the classical pathway using NLS within Rpt2, Rpn2, and Stst1. Ump1 and Sts1 are short-lived and degraded with the completion of proteasome maturation and the assembly of holo-enzymes in the nucleus.

A few years later, CP biogenesis became a major topic of interest. Despite the short half-life, CP precursor complexes resembling a half-CP were detectable due to genetic manipulations in yeast [72] and could be traced by the maturation factor Ump1 [44]. Three major observations led us to hypothesise that half-CP are imported into the nucleus of dividing yeast cells (Figure 2). First, Ump1 can be functionally tagged with GFP and is predominantly nuclear, although the half-CP is assembled from newly synthesised subunits in the cytoplasm. Thus, we concluded that the CP is imported into the nucleus as an immature protease. Second, the CP was mislocalised to the cytoplasm in Ran cycling and importin α mutants, specifically in the *srp1-49* (E145K) mutant but not in the *srp1-31* (S166F) mutant, suggesting that the canonical pathway is responsible for the nuclear import of nascent proteasomes [73].

Recent studies confirmed that nuclear localisation of proteasomes is disturbed in the *srp1-49* mutant but not in the *srp1-31* mutant, while the nuclear import of commonly used cNLS reporter proteins is affected in *srp1-31* but not in *srp1-49* mutants. These observations were initially disconcerting, but suggested that proteasomal NLS are differentially recognised than cNLS prototypes by Srp1 [74] and confirmed early studies which suggested an additional role for Srp1 in the regulation of protein degradation separate from its well-established role as NLS receptor [75]. Srp1 was originally identified as a suppressor of certain temperature-sensitive (ts) mutants in RNA polymerase I (Pol I) in *Saccharomyces cerevisiae*, and different ts mutants in *SRP1* displayed allele-specific phenotypes which might be consequences of defective nuclear import [76].

Our work revealed that Srp1 is co-immunoprecipitated with CP precursor complexes and not mature CP as demonstrated by the presence of unprocessed and incompletely processed CP subunit β5, crucial determinants of CP maturation [73]. The presence of incompletely processed β5 subunits suggests that CP maturation occurs with nuclear transport, since incompletely processed β5 is indicative of the pre-holo-CP.

Third, most CP is found to be nuclear when CP maturation is severely delayed by *UMP1* deletion [77]. This observation supports the conclusion that the majority of the CP matures in the nucleus [73]. The deletion of *UMP1* results in the reduced efficiency of CP maturation by approximately 50%, which is compensated for by doubling the amount of CP precursor complexes [42]. If the majority of the CP were matured in the cytoplasm, one would expect a significant increase of GFP-labelled β5 in the cytoplasm. The opposite is observed in *ump1Δ* cells expressing GFP-labelled β5. Additionally, other proteasomal reporter proteins remain primarily nuclear in *ump1Δ* cells, indicating nuclear accumulation of inactive and mature CP [77].

Although fluorogenic substrates either microinjected into the nucleus of mammalian cells or soaked by yeast spheroblasts are cleaved *in situ* in the nuclear periphery, the fraction of nuclear proteasomes engaged in protein degradation was not determined [10,13].

We sought to investigate how the nuclear import of CP precursor complexes by importin αβ is related to Tanaka’s hypothesis of import-competent CP. Recent single particle cryo-electron microscopy studies revealed that CP precursor complexes undergo significant conformational changes during CP maturation. The α- and β-rings are rather flexible in CP precursor complexes [46], while the α-ring gates are tightened in the mature CP [47]. Based on these structural data, it is plausible to assume that the cNLS are accessible in CP precursor complexes and masked in mature CP, particularly when mature CP associates with the RP to yield holo-enzymes with RP-CP-RP configuration.

Next, we will summarise our current knowledge on nuclear import of the RP lid and base.

#### 2.4.2. Nuclear Import of the RP

To deliver proteasomal subcomplexes in similar stoichiometry into the nucleus, it is conceivable to assume that the same pathway would be taken for the nuclear import of proteasomal subcomplexes (Figure 2). Thus, Wendler *et al.* investigated whether the canonical import pathway also applied to the nuclear import of the RP [78]. First, *srp1-31* and *srp1-49* mutants were tested for mislocalisations of GFP-labelled Rpn1 and Rpn11, reporters of RP base and lid, respectively. Consistent with the localisation studies on the CP, GFP-labelled Rpn1 and Rpn11 mislocalised to the cytoplasm in temperature-sensitive *srp1-49* cells, as later confirmed by others [74]. All available yeast mutants with deficiencies in importins were tested for proteasome mislocalisations. Notably, most of these importins are not essential. Compared with wild-type, none of the non-essential importins showed cytoplasmic mislocalisations of proteasomes (Wendler and Enenkel, unpublished work). We inferred that an essential nuclear cargo such as the proteasome would require an import pathway mediated by an essential importin such as Srp1/Kap95. Based on these observations, Wendler *et al.* searched for putative cNLS motifs within RP subunits. The N-terminal region of Rpt2 and the C-terminal region of Rpn2, both subunits of the RP base, harbour putative cNLS. Fused to GFP, which itself is not targeted to the nucleus, the Rpt2 and Rpn2 NLSs directed the fusion protein into the yeast nucleus and were recognised by importin αβ, showing their functionality in nuclear import. The study also aimed to verify the functionality of the Rpt2 and Rpn2 NLSs in the context of the RP base. Compared to wild-type RP base, RP base truncated in either Rpt2 or Rpn2 NLS was not recognised by importin αβ. The Rpt2 NLS but not the Rpn2 NLS was dispensable for nuclear targeting. The deletion of the Rpn2 NLS led to a ts phenotype with the mislocalisation of the RP base in cytosolic foci and cell cycle arrest at the restrictive temperature [78]. In the following years, Isono *et al.* confirmed that Rpn2 contributes an essential NLS to nuclear import of the RP base and that the RP lid is imported into the nucleus independently of the RP base, suggesting that nuclear proteasomes are assembled from subcomplexes [79]. Intriguingly, in *Xenopus* eggs, Rpn2 and Kap95 were identified by mass spectrometry within an import-competent intermediate of proteasome assembly, providing yet another link between nuclear import and assembly of proteasome holo-enzymes [80]. Both Rpn2 and Kap95 [60] suggest synergistic functions in nuclear import.

In contrast to the CP and the RP base, no putative cNLS could be identified within the RP lid subunits. Instead, the RP lid subunit Rpn11 interacts with the NLS-containing protein Sts1 by yeast two-hybrid. Since *RPN11* and *STS1* are high dosage suppressors of the *srp1-49* mutation and Sts1 directly interacts with Srp1 [75], Sts1 most likely mediates the interaction of the RP lid with Srp1. Intriguingly, the deletion of the NLS of Sts1 (*sts1ΔNLS*) prevented not only the interaction between the RP lid and Srp1 by causing cytoplasmic retention of the RP lid, but also interfered with the nuclear import of the RP base and the CP, suggesting that Sts1 has a global impact on the nuclear localisation of proteasome holo-enzymes, which cannot be compensated for by other NLSs within proteasomal subunits [81]. Sts1 is essential in budding yeast and has an ortholog in fission yeast named Cut8 [82]. Cut8 was proposed to anchor proteasomes to the nuclear side of the NE [83,84]. However, its extremely short half-life and absence in higher eukaryotes disputes its role as a scaffold protein involved in embedding proteasomes into the NE.

Over the last two decades, the cytoplasmic retention of proteasomes, failures in the degradation of polyubiquitylated proteins and of cyclin Clb2 followed by cell cycle arrest, was ascribed to mutations in Srp1 [74,75,85]. Fink and his coworkers were the first to report on Srp1 function in mitosis [85], which according to our present knowledge is due to its function in the nuclear import of proteasomes. Their studies were based on the *srp1-31* mutant, while Tabb *et al.*, Chen *et al.*, and we attributed the phenotype of aberrant proteasome function and localisation to the *srp1-49* mutant [70,71]. Here, it is appropriate to clarify these discrepancies in the literature. We studied *srp1* mutants from Fink’s and Nomura’s laboratories, respectively. We found that Fink’s *srp1-31* and *srp1-49* mutants were mixed after we mapped the mutations and compared them with the original mutations from Nomura’s lab. Thus, the original studies on Srp1 by Loeb *et al.* [81] are consistent with later studies by Tabb *et al.* and Chen *et al.* [70,71].

#### 2.4.3. Nuclear Import of the Proteasome Holo-Enzymes

So far, the nuclear import processes described above took as their basis the model that favours the translocation of proteasomes in modules, namely CP precursor complexes, the RP base and lid. After being individually imported into the nucleus via the canonical importin/karyopherin αβ pathway, in the presence of proteasome-dedicated chaperones such as Ump1, the final step of nuclear proteasome assembly could take place in the nucleus [73,78,79]. However, recent studies based on quantitative live cell imaging suggested that proteasome holo-enzymes could also complete their assembly in the cytoplasm and shuttle through the NPC [64]. To address the question around the mobility of proteasome holo-enymes, Pack *et al.* conducted fluorescence correlation spectroscopy (FCS) in living yeast cells to determine the absolute concentration, spatio-temporal dynamics, and assembly of proteasome holo-enzymes. First, Pack *et al.* analysed the dynamics of the CP and RP base and lid at their endogenous expression levels in exponentially growing yeast cells. The distribution of chromosomally GFP-tagged Pre6 (CP subunit α4), Rpn1 (RP base), and Rpn7 (RP lid) in the nucleus and cytoplasm was monitored. The FCS curves showed a similar distribution in both compartments for all three subunits reflecting equal stoichiometry of the CP and the RP base and lid, with 140–200 nM in the cytoplasm and 830–980 nM in the nucleus. In the second part of their study, Pack *et al.* demonstrated by dual colour fluorescence cross correlation spectroscopy (FCCS) that proteasome holo-enzymes are stable in living cells. Compared with wild-type cells, the proteasome concentration is increased two-fold in the cytoplasm and decreased three-fold in the nucleoplasm of *srp1-49* mutants, suggesting that their nuclear import depends on Srp1, possibly governed by Sts1. However, the detection of solely RP-CP-RP and no proteasomal subcomplexes contradicted previous native PAGE analysis using multiplex imaging by which considerable amounts of free CP and RP subcomplexes were detected [38,86]. One possibility is that proteasome holo-enzymes dissociate upon cell disintegration, especially if CP and RP are labelled with differently coloured fluorescent proteins. Additionally, CP precursor complexes and pre-holo-CP were not detectable by FCS due to their low abundance. To identify them by biochemical means, they need to be stabilised, e.g., by GFP-tagged versions of Ump1 or Pre6 (CP subunit α4), which delay β5 pro-peptide processing so that even incompletely processed β5 become detectable [73]. Pack *et al.* did not analyse the maturation state of the proteasomes carrying the GFP-tagged CP subunit α4 and may have monitored a mixture of mature CP and pre-holo-CP. Consequently, pre-holo-CP could be the *de facto* assembly state at which nascent CP is imported into the nucleus and not the CP precursor complex resembling the half-CP (Figure 2). Half-CPs identified as import cargoes in previous biochemical approaches could be decay products of pre-holo-CPs, which are highly unstable upon cell disintegration. Considering these limitations of biochemical approaches *versus* non-invasive FCS–FCCS technologies, Pack *et al.* did not exclude the possibility that CP assembly intermediates are transported by the canonical pathway [64].

**Figure 3:**
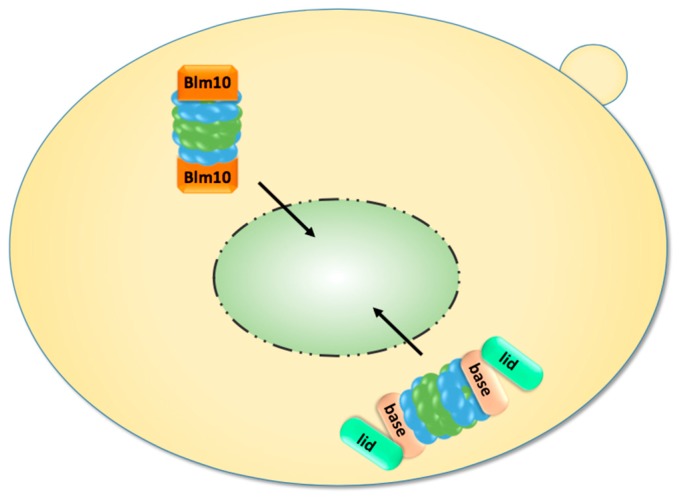
Upon exit from quiescence, mature proteasomes are imported into the nucleus. They are either imported as holo-enzymes or by Blm10, which facilitates the nuclear import of mature CP.

Finally, genetically tethered RP-CP-RP were analysed by FCS. Since it was difficult to decide whether they are assembled in the cytoplasm or nucleoplasm, Pack *et al.* examined yeast cells grown to stationary phase in which proteasomes accumulate in cytoplasmic storage granules. Upon exit from quiescence, the proteasome granules rapidly cleared and genetically tethered RP-CP-RP were detected in the nucleus, providing further evidence that assembled proteasome RP-CP-RP can pass through the NPC (Figure 3) [64].

Since the synthesis of new proteins is stalled during quiescence, young CP precursor complexes are not available as import cargoes. Thus, an alternative import pathway must exist that makes use of older matured proteasomes stored in proteasome granules during quiescence, as outlined next [31,64].

### 2.5. Nuclear Import of Proteasomes upon Exit from Quiescence

As mentioned in the last section, proteasomes are exported from the nucleus into the cytoplasm with the transition from proliferation to quiescence. Proteasome export is a poorly understood area and we will not stress on any speculations about its mechanism. Interestingly, in prolonged quiescence ~90% of the proteasomes are found in membraneless spherical organelles called proteasome storage granules (PSG), which migrate through the cytoplasm in yeast [15]. Upon exit from quiescence, the PSG rapidly clear and proteasomes are delivered to the nucleus within a few minutes, a time frame which does not allow the assembly of new precursor complexes and RP modules. In order to resume cell growth, proteasomal proteolysis of short-lived nuclear proteins regulating cell cycle progression must be recommenced by old matured proteasomes which are released from the PSG. Here, it is important to note that proteasome holo-enzymes are less stable in quiescence, since they dissociate into RP and CP upon cell disintegration. If RP-CP disassembly is not an artefact of biochemical fractionation, RP and CP might not be physically associated during quiescence even though they localise within the PSG. The propensity of RP-CP-RP to dissociate in quiescence is in line with the decline of ATP and the reduced engagement of proteasomes in the degradation of polyubiquitylated proteins [87]. Notably, in quiescent yeast, most CP is associated with Blm10, a conserved 240 kDa protein of the HEAT repeat-like family, and not with the RP [31].

We found that Blm10 represents the first characterised CP-dedicated nuclear transporter receptor which facilitates the nuclear import of mature CP (Figure 3) [31]. This conclusion was drawn from several experimental pieces of evidence. First, quiescent *blm10Δ* mutants significantly lag in resuming cell growth because nuclear resumption of the CP to the nucleus is delayed for about two hours relative to wild-type cells, a time frame which is required to provide newly synthesised CP precursor complexes as import cargoes. Another piece of evidence is that Blm10 binds FG-rich nucleoporins and GTP-bound Gsp1, the yeast ortholog of the small GTPase Ran. Blm10 is also dissociated from the CP by Ran-GTP, suggesting that Blm10 behaves like Kap95, the canonical importin β. Strikingly, Blm10 and importin β show similar α-solenoid folds within certain HEAT repeat proteins [60]. Although Blm10 is mainly associated with a fraction of mature CP [88], it is also part of incompletely matured pre-holo-CP and CP precursor complexes [89]. However, the fraction of Blm10-associated CP precursor complexes is significantly less abundant than the fraction of CP precursor complexes. Blm10-associated CP precursor complexes are only detectable in highly proliferating yeast cells if CP precursor complexes are stabilised by tagged versions of Ump1 or the deletion of *UMP1* [43,77].

Using this genetic approach, we could show that Blm10 is preferentially associated with constitutively open or disordered α-rings, suggesting a role in the control of proper α-ring gating during CP maturation [77,89]. With regards to Blm10’s role in nuclear import, the fraction of Blm10-associated CP precursor complexes most likely represents alternative import intermediates that bypass the canonical pathway (Figure 2). Both the Blm10- and importin αβ-dependent proteasomal cargoes share a common feature. Their α-rings are in an open conformation and accommodate an import-competent state of the CP. It still remains to be solved how the RP is imported into the nucleus upon exit from quiescence.

Rpn2, a RP base subunit with a similar α-solenoid fold as found for importin β and also belonging to the family of HEAT-repeat proteins [60,90], could be a candidate for a nuclear import receptor for the RP. Even the hybrid of Blm10-associated RP-CP could serve as an import carrier.

Evidently, future studies are required to clarify which nuclear import pathway predominates within the cell by using defined growth conditions.

### 2.6. Nuclear Export of Proteasomes

Post-translational modifications, such as phosphorylation, have been reported to regulate the activity of nuclear proteasomes [91]. Post-translational modifications also affect proteasome dynamics. For example, *N*-myristoylation of Rpt2 contributes to nuclear proteasome localisation [92]. Disturbed *N*-acetylation of yet unknown proteasomal targets results in the deficient nuclear export of proteasomes and, thus, the impaired formation of proteasome granules in quiescence [32]. Thus, it is not surprising that multiple factors will orchestrate in proteasome dynamics. Another key question to be answered in the future is why proteasomes are exported from the nucleus into the cytoplasm during quiescence.

## 3. Conclusions

In this review, we have summarised the literature covering the nuclear import of yeast proteasomes. Since only a few *in vitro* studies using reconstitution systems for mammalian proteasomes are known from the 1990s, we restricted our discussions to the nuclear import pathways of yeast proteasomes. The results from yeast will markedly extend our knowledge of proteasome dynamics, protein degradation, and spatial quality control in mammalian cells, especially during transitions between proliferation and quiescence, which is accompanied by a high traffic volume of proteasomes and possible substrates between the nucleoplasm and cytoplasm.

A long-term goal is to gain a further understanding of metabolic changes that force proteasomes into granules, that is, why proteasome granules are exported from the nucleus and stored in the cytoplasm. Proteasome granules, which serve cytoprotective functions, are kept on call to respond to extracellular signals and to be reactivated for nuclear import. Clarifying the open questions around the nuclear transport of proteasomes has implications for cellular stress responses and defence mechanisms in the aging of non-dividing cells in humans.
